# Is Serum BDNF Altered in Acute, Short- and Long-Term Recovered Restrictive Type Anorexia Nervosa?

**DOI:** 10.3390/nu13020432

**Published:** 2021-01-29

**Authors:** Jonas L. Steinhäuser, Joseph A. King, Friederike I. Tam, Maria Seidel, Ronald Biemann, Marie-Louis Wronski, Daniel Geisler, Veit Roessner, Stefan Ehrlich

**Affiliations:** 1 Division of Psychological and Social Medicine and Developmental Neurosciences, Faculty of Medicine, Technische Universität Dresden, 01307 Dresden, Germany; Jonas.Steinhaeuser@uniklinikum-dresden.de (J.L.S.); Joseph.King@uniklinikum-dresden.de (J.A.K.); Friederike.Tam@uniklinikum-dresden.de (F.I.T.); Maria.Seidel@uniklinikum-dresden.de (M.S.); Marie-Louis.Wronski@uniklinikum-dresden.de (M.-L.W.); Daniel.Geisler@uniklinikum-dresden.de (D.G.); 2 Eating Disorder Treatment and Research Center, Department of Child and Adolescent Psychiatry, Faculty of Medicine, Technische Universität Dresden, 01307 Dresden, Germany; 3 Institute of Laboratory Medicine, Clinical Chemistry and Molecular Diagnostics, University of Leipzig, 04103 Leipzig, Germany; Ronald.Biemann@medizin.uni-leipzig.de; 4 Institute for Clinical Chemistry and Pathobiochemistry, Otto-Von-Guericke University Magdeburg, 39120 Magdeburg, Germany; 5 Department of Child and Adolescent Psychiatry, Faculty of Medicine, Technische Universität Dresden, 01307 Dresden, Germany; Veit.Roessner@uniklinikum-dresden.de

**Keywords:** brain-derived neurotrophic factor, BDNF, eating disorders, anorexia nervosa, food intake regulation, restrictive eating, endocrinology, neuroendocrinology, biomarkers, neuromarkers

## Abstract

Brain-derived neurotrophic factor (BDNF), a neurotrophin involved in the regulation of food intake and body weight, has been implicated in the development and maintenance of Anorexia nervosa (AN). The majority of previous studies reported lower BDNF levels in acutely underweight AN patients (acAN) and increasing levels after weight rehabilitation. Here, we investigated serum BDNF concentrations in the largest known AN sample to date, both before and after weight restoration therapy. Serum BDNF was measured in 259 female volunteers: 77 in-patient acAN participants of the restrictive type (47 reassessed after short-term weight rehabilitation), 62 individuals long-term recovered from AN, and 120 healthy controls. We validated our findings in a post-hoc mega-analysis in which we reanalyzed combined data from the current sample and those from our previous study on BDNF in AN (combined sample: 389 participants). All analyses carefully accounted for known determinants of BDNF (age, sex, storage time of blood samples). We further assessed relationships with relevant clinical variables (body-mass-index, physical activity, symptoms). Contrary to our hypotheses, we found zero significant differences in either cross-sectional or longitudinal comparisons and no significant relationships with clinical variables. Together, our study suggests that BDNF may not be a reliable state- or trait-marker in AN after all.

## 1. Introduction

Anorexia nervosa (AN) is a life-threatening eating disorder characterized by body image disturbances and an intense fear of weight gain which motivate extreme energy-intake restriction, resulting in significantly low body weight and serious medical complications. The disorder affects women more frequently than men (8:1 ratio), typically develops during adolescence and has the highest standardized mortality rate in psychiatry [1,2]. AN can be categorized clinically into one of two types: a restricting-type (AN-R), which is defined by severe food restriction, and a binge-eating/purging-type (AN-BP), which is characterized by a large intake of calories in a short amount of time (bingeing), followed by measures to prohibit absorption of nutrients (purging, e.g., vomiting or abuse of laxatives) in order to prevent weight gain [3]. The etiology and pathophysiology are complex and a better understanding of the underlying biological, psychological and sociocultural factors influencing disorder onset and maintenance is essential to developing more effective treatments [4].

One biological factor implicated in AN pathogenesis is dysfunction in the physiology of certain central and peripheral regulatory proteins involved in modulating appetitive behavior [5,6,7,8,9], including leptin, ghrelin, peptide YY, agouti-related protein, endocannabinoids, and brain-derived neurotropic factor (BDNF). Regarding BDNF, a neurotrophin responsible for synaptic plasticity as well as the development, function and survival of neurons [10,11,12], this growth factor is generally hypothesized to play an important role in several neuropsychiatric conditions [13,14,15,16], including other eating disorders [17,18]. Much pre-clinical evidence supports a link between BDNF and a range of functions relevant to the development and maintenance of AN. For example, a role in food intake regulation is well-established [19,20], and nutritional status and choice of diet are known to influence BDNF levels in both animals [21] and humans [22,23]. Further, the involvement of BDNF in altering eating behavior is supported by the discovery of BDNF-expressing neurons in the paraventricular hippocampus that regulate energy intake, thermogenesis, and physical activity [24]. BDNF is also expressed in brain regions belonging to and connecting with the mesolimbic pathway, associated with functions such as reward-related processing and learning [25,26,27]; domains reported to be aberrant in AN [28,29,30,31].

In line with the aforementioned relationships between BDNF and functions relevant to AN, a growing number of studies have reported BDNF alterations in AN patients [18,32,33,34,35]. The first evidence for a role of BDNF in AN came from Nakazato et al. [18], who reported significantly lower serum BDNF levels in a small sample of individuals (aged 14–34 years) with acute AN (acAN) compared to both healthy comparison participants (HC) and individuals with bulimia nervosa (BN). Additionally, studies have shown relationships between BDNF and eating behavior in AN-R [33] as well as significantly lower BDNF levels in AN-R than in AN-BP [6]. The main finding of decreased BDNF levels in acAN has now been repeatedly evaluated and mostly confirmed [17,32,34,35,36,37], suggesting that lower BDNF levels may represent an adaptive phenomenon of the body to increase food intake and achieve metabolic homeostasis in the undernourished individual [8]. However, some studies have reported inconclusive results [6,38,39]. Moreover, it remains unclear whether decreased BDNF in acAN may merely reflect a consequence of malnourishment as relatively few studies have investigated BDNF levels longitudinally during weight restoration treatment or in long-term weight-recovered individuals with a history of AN (recAN). While Nakazato et al. [40] did not observe differences in BDNF levels after partial weight rehabilitation, others have found a significant increase [35,39]. To our knowledge, all studies examining BDNF concentrations between recAN and HC did not detect any persistent group differences [39,41,42]. Nonetheless, interpretation of many of these prior results might be limited due to relatively small sample sizes and challenges in accounting for factors influencing BDNF levels (e.g., age, time of blood draw, storage time of samples) [32,43].

In addition to the need for statistically higher-powered studies in individuals with AN both during acute illness and across weight restoration therapy, there are several important methodological considerations with respect to the investigation of BDNF that have only partially been accounted for in previous studies. First, serum and plasma BDNF may represent two different pools of BDNF [44,45], and measuring BDNF concentrations in serum has been argued to be advantageous due to critical dependencies of plasma BDNF concentrations on pre-analytic strategies (e.g., choice of anticoagulant, pre-analytic storage temperature) [46,47]. Second, several studies in healthy individuals report an association between physical exercise and BDNF levels: while the majority of studies indicate an increase in BDNF levels after acute exercise, results vary regarding alterations of basal BDNF levels induced by regular exercise, with smaller effect sizes reported in females (for reviews see, e.g., [48,49]). Third, the time between processing and storage of blood samples and the measurement of BDNF levels is known to be influential, with BDNF levels in serum being stable stored up to twelve months [50] and decreasing significantly after that [51].

Given the importance of distinguishing between state- and trait-markers in studying AN [29,52,53], the current study examined both longitudinal changes in BDNF concentrations before and after partial weight restoration as well as cross-sectionally in acAN and recAN in comparison to HC. Our analyses focused on acAN and recAN of the restrictive type to render our results more comparable to findings from the aforementioned previous studies that reported alterations in BDNF only in AN-R, but not AN-BP. Additionally, focusing on AN-R allowed us to validate the influence of BDNF levels on food intake regulation since eating patterns differ drastically in AN-R and AN-BP. We studied serum-derived BDNF concentrations in the largest female sample of acAN (both longitudinally and cross-sectionally) and recAN to date, while controlling for the influence of storage time, physical exercise, age, and batch effects. To verify the results, we additionally reanalyzed data from our previous study on serum BDNF in AN [39] in a post-hoc mega-analysis, pooling data from both studies.

To foreshadow our findings, in contrast to previous reports of lower BDNF concentrations in acAN and relative normalization across weight recovery, we found zero significant effects, suggesting that BDNF may not be a robust marker of either state or trait in AN after all.

## 2. Materials and Methods 

### 2.1. Participants

The sample at the focus of the current study consisted of a total of 259 female volunteers: 77 acAN of the restrictive type (12–29 years old); 62 recAN, formerly diagnosed with AN-R (15–29 years old); and 120 HC (12–29 years old). All participants (or their legal guardians if participants were younger than 18 years old) provided written informed consent. The study was conducted according to the guidelines of the Declaration of Helsinki and approved by the Institutional Review Board of Technische Universität Dresden (EK 14012011, date: 6 February 2013).

Current and past diagnoses of AN according to the Diagnostic and Statistical Manual of Mental Disorders (DSM-V) [3] were explored in all participants and pertinent information on possible confounding variables (e.g., substance abuse, bulimic symptoms) were collected using the expert form of the Structured Interview for Anorexia and Bulimia Nervosa (SIAB-EX) [54] as well as our own semi-structured interview (e.g., medication) and medical records.

Inclusion criteria for acAN required a body mass index (BMI) below the 10th age percentile (for individuals younger than 15.5 years) or below 17.5 kg/m² (for individuals older than 15.5 years). AcAN participants were admitted to a specialized eating disorder program of the University Hospital Carl Gustav Carus at the TU Dresden and were assessed within 96 h after the initiation of intensive treatment (acAN-T1, study timepoint T1). Forty-seven of the 77 acAN patients were reassessed after partial/short-term weight rehabilitation (acAN-T2, study timepoint T2) with a BMI increase of at least 10% as in previous longitudinal studies from our group [55,56]. In the current sample, all acAN had a ≥13% BMI increase at follow-up.

Inclusion criteria for recAN comprised: (1) diagnosis of AN in the past, (2) a BMI above the 10th age percentile (for individuals younger than 18 years) or above 18.5 kg/m² (for individuals older than 18 years) for at least 6 months prior to the study, (3) menstruation, and (4) the absence of binging, purging, or engaging in substantial restrictive eating patterns. To be included in this study, HC had to be of normal weight (BMI ≥ 15th age percentile and ≤ 85th age percentile for individuals younger than 18 years, BMI ≥ 18.5 and ≤ 25 for individuals older than 18 years), eumenorrheic, and without any history of psychiatric illnesses, as assessed with the SIAB-EX and our in-house semi-structured interview which includes questions regarding exploring general psychopathology. HC were recruited through advertisement among middle school, high school, and university students.

For all groups, additional exclusion criteria including, most importantly, any history of binge eating or bulimia nervosa, substance dependence or abuse, and neurological or medical conditions were applied. Further, history of any of the following diagnoses led to exclusion from this study for participants of all groups: organic brain syndrome, schizophrenia, psychosis not otherwise specified, or bipolar disorder. Further exclusion criteria for all participants were an IQ below 85; inflammatory, neurologic, or metabolic illness; chronic medical or neurological illness that could affect appetite, eating behavior or body weight; clinically relevant anemia; pregnancy or breast feeding. Due to the requirements of our greater ongoing study in AN, psychotropic medication within 4 weeks before the study (except for selective serotonin reuptake inhibitors) was an additional exclusion criterion for all groups.

To supplement the partially unanticipated null results obtained from the sample described above, we additionally included—in a post-hoc mega-analysis—all data from participants who were recruited from Site A (Berlin) in our previous multicenter study [39]: 55 acAN (14 reassessed after partial/short-term weight rehabilitation), 23 recAN, and 52 HC; resulting in a total combined sample of 389 female volunteers: 132 acAN (61 reassessed after partial/short-term weight rehabilitation), 85 recAN, and 172 HC. Demographic information for participants included in the mega-analysis as well as details regarding nominal differences in inclusion criteria and study procedures (e.g., specific sandwich enzyme-linked immunosorbent assay (ELISA) kit) used in Zwipp et al. [39] relative to our current study are provided in the Supplementary Material Tables S1 and S2. Please refer to Zwipp et al. for full details on procedures and participants of our previous study [39].

### 2.2. Clinical Assessment

Information gained from the SIAB-EX interview was complemented by the German version of the self-report questionnaire Eating Disorder Inventory-2 (EDI-2) [57] and the German version of the Beck Depression Inventory-II (BDI-II) [58]. Intelligence quotient (IQ) was estimated using short versions of German adaptions of the Wechsler Adult Intelligence Scale [59], or the Wechsler Intelligence Scale for Children [60] if participants were aged 15 years or younger. Participants’ physical activity within three months prior to the study was assessed using the SIAB-EX, as described in detail in Holtkamp et al. and Ehrlich et al. [61,62]. The SIAB-EX quantifies the intensity and frequency of physical activity within the last three months on an ordinal scale with five levels. First level (0): no excess physical activity; Second level (1): excess physical activity up to 2× per week; Third level (2): excess physical activity at least 2× per week; Fourth level (3): excess physical activity up to 1× per day; Fifth level (4): excess physical activity multiple times a day. To account for height and weight changes during adolescence, BMI standard deviation scores (BMI-SDS) [63,64] were calculated. All study data were entered and managed using the secure, web-based electronic data capture tool REDCap (Research Electronic Data Capture) [65].

### 2.3. BDNF Measurements

Venous blood samples were collected into vacutainer tubes between 7 and 9 a.m. after an overnight fast. To yield blood serum, blood samples were left to clot for 30 min at 6–8 °C and then centrifuged (800 g for 15 min) in a pre-cooled (5 °C) centrifuge. The samples were then aliquoted into pre-cooled Eppendorf Tubes^®^ and stored at −80 °C. The protocol for obtaining and processing serum in the current study was identical to that in previous studies from our group [39,66,67]. Free serum BDNF concentrations were quantified using commercially available ELISA kits according to the manufacturer’s instructions (R&D Systems, Minneapolis, MN). Blood samples were processed in three batches. The first batch was measured in duplicate, yielding an intra-assay coefficient of variation (CV) of 4.6%. As this was in line with the manufacturer’s specifications (intra-assay CV: 3.8–6.2%, inter-assay CV: 7.6–11.3%), serum samples in the second and third batch were analyzed only once. For the first batch, the mean concentrations from our duplicate measurements were used in all statistical analyses. All batches were analyzed by the same laboratory.

### 2.4. Statistical Analyses

AcAN and recAN were individually age-matched to HC by means of an automated search algorithm for optimal pairs [68] to account for the influence of age on serum BDNF concentration [50], resulting in two cohorts labelled, respectively, in the following as acAN-HC_acAN_ and as recAN-HC_recAN_. Data of 19 HC were used in both cross-sectional comparisons. Group comparisons on clinical and demographic data were carried out using independent-samples *t*-tests for the cross-sectional, and paired-samples *t*-tests for the longitudinal comparisons, respectively. Because of the known influence of storage time of blood samples on BDNF concentrations [43,51], we assessed these relationships in our data (using both regular and squared storage time). Based on significant results (see Supplementary Figures S1 and S2), we aimed to control for undue influence of storage time as well as potential batch effects by employing a multiple linear regression model predicting serum BDNF concentrations based on storage time, squared storage time and batch in each study sample separately. Each sample’s overall mean was added back to the unstandardized residuals from the model to improve interpretability of BDNF concentrations. As in our previous study, residualized serum BDNF concentrations were used in all further analyses [39]. Normal distributions of BDNF concentrations in all groups were confirmed by visual inspection of histograms and a kernel density estimate. In order to examine whether the study groups differed in BDNF concentrations, we employed independent samples *t*-tests for the cross-sectional comparisons and paired-samples *t*-tests for the longitudinal comparison, respectively. Additional exploratory analyses assessed potential group differences between the acAN and the recAN group using a multiple linear regression model, controlling for participants’ age, since age-matching between these groups was not possible in our sample. Motivated by the results suggestive of “absence of evidence” (i.e., no significant group differences in BDNF concentrations) obtained from these analyses within the traditional null-hypothesis significance testing (NHST) framework, we also carried out supplementary analyses using Bayesian inference to assess whether our data actually provide “evidence of absence” [69,70].

Potential relationships between serum BDNF levels and clinical variables (BMI-SDS, EDI-2, BDI-II, and physical activity) were assessed using Pearson’s product-moment or Spearman’s rank correlation for the total sample as well as for each of the groups separately.

For the follow-up mega-analysis, we tested for group differences in serum BDNF concentrations in the combined cross-sectional sample using a multiple regression approach with planned contrasts while controlling for participants’ age and adding a dummy variable indicating whether subjects were a part of the sample compiled for the current study or whether they were originally included in our previous study [39]. For the combined longitudinal sample, a mixed-effects model was applied, including the same dummy variable as in the cross-sectional analysis.

Statistical analyses using traditional NHST were conducted using the R Software package [71]. Plots, tables and statistical reports were created using the ggplot2 [72], stargazer [73] and report [74] packages as well as the raincloud plot function [75]. Bayesian test statistics were calculated using JASP 0.14 [76] (detailed information on JASP settings used are provided in the Supplementary Material).

## 3. Results

### 3.1. Sample Characteristics

Clinical and demographic characteristics of the study sample are summarized in Table 1 for the cross-sectional comparison and in Table 2 for the longitudinal comparison. As expected, acAN participants had a lower BMI and higher symptom scores than their healthy counterparts.

The recAN-HC_recAN_ comparison revealed persistently lower BMI in recAN, but values were nonetheless in the normal range. Significantly higher symptom scores in recAN indicate elevated residual symptoms despite long-term weight recovery.

In the longitudinal sample, the anticipated significant increase in BMI and general improvement in symptom scores after partial weight restoration was observed.

Regarding the participants from Site A (Berlin) of our previous study that were included in the post-hoc mega-analysis, acAN presented with lower BMI and higher symptom scores than HC as well. RecAN participants similarly had lower BMI than their healthy counterparts, but were in the normal range nonetheless. However, recAN participants were older and did not differ on symptom scores compared to HC (Supplementary Table S1). Longitudinally, BMI increased significantly as expected, and symptom scores were not reevaluated in those participants (Supplementary Table S2).

### 3.2. Comparisons of Serum BDNF Concentrations

**Figure 1:**
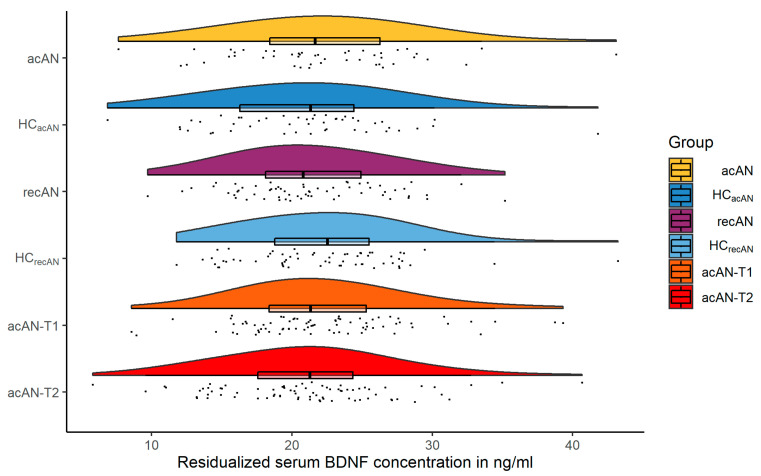
Raincloud plots (distributions, individual data points and box plots) of residualized serum BDNF concentrations in ng/mL for all three comparisons. acAN and recAN groups are shown with their respective age-matched HCs. The longitudinal acAN sample is presented as acAN-T1 and acAN-T2. Abbreviations: *BDNF* brain-derived neurotrophic factor; *acAN* acute anorexia nervosa participants; *HC_acAN_* healthy control participants, age-matched to the acAN group; *recAN* long-term recovered anorexia nervosa participants; *HC_recAN_* healthy control participants, age-matched to the recAN group; *acAN-T1* acute anorexia nervosa participants at study timepoint 1 (admission); *acAN-T2* acute anorexia nervosa participants at study timepoint 2 (after short-term weight rehabilitation).

A visual summarization of the main results is presented in Figure 1. The difference in serum BDNF concentrations by group (mean in group HC_acAN_ = 22.18 ng/mL, mean in group acAN = 21.01 ng/mL) was numerically small and statistically not significant using traditional NHST (difference = −1.17 ng/mL, 95% CI [−0.71, 3.05], Welch Two Sample *t*-test t(151.73) = 1.23, *p* = 0.221; Cohen’s d = 0.20, 95% CI [−0.12, 0.52]), while Bayesian analysis delivered anecdotal evidence in support of the null-hypothesis (BF_10_ = 0.348, Supplementary Figure S3). 

In the recAN-HC_recAN_ sample, the same model (mean in group HC_recAN_ = 21.34 ng/mL, mean in group recAN = 22.17 ng/mL) also revealed a numerically small and statistically not significant group difference (difference = 0.83 ng/mL, 95% CI [−2.72, 1.06], t(121.24) = −0.87, *p* = 0.386; Cohen’s d = −0.16, 95% CI [−0.51, 0.20]), with Bayesian analysis providing moderate evidence in favor of the null-hypothesis (BF_10_ = 0.27, Supplementary Figure S4). 

The results of the longitudinal comparison (mean in group acAN-T2 = 22.20 ng/mL, mean in group acAN-T1 = 20.91 ng/mL) showed similarly small and statistically not significant differences (mean of the differences = −1.29 ng/mL, 95% CI [−2.92, 0.34], paired *t*-test t(46) = −1.59, *p* = 0.118; Cohen’s d = −0.23, 95% CI [−0.53, 0.06]), with Bayesian analysis indicating anecdotal evidence for the null-hypothesis (BF_10_ = 0.512, Supplementary Figure S5). Confirmatory results were obtained using an alternative mixed model statistical approach (Supplementary Tables S8 and S9) and by calculating the type II error of our original model (Supplementary Material).

These results remained unaffected by excluding participants who received treatment with selective serotonin reuptake inhibitors within 4 weeks before blood sample collection (Supplementary Material).

Additional exploratory comparisons between serum BDNF concentrations of the acAN and the recAN group also did not provide any evidence for significant between-group differences (Supplementary Tables S10 and S11).

The results of correlation analyses indicated no significant association between serum BDNF concentrations and BMI-SDS, EDI-2 total, BDI-II, or physical activity in neither the total sample, nor in any of the groups individually, even without correction for multiple comparisons (Supplementary Table S3).

The results from our supplementary post-hoc mega-analysis including data originally analyzed in our previous study [39] amplified the main findings outlined above; indicating no significant group differences between acAN and HC, recAN and HC, and acAN before and after partial/short-term weight rehabilitation, respectively (Supplementary Tables S4 and S5). These results were robust and remained unaffected after excluding acAN and recAN of the binge-purge clinical subtype who were included in our previous study (Supplementary Tables S6 and S7).

## 4. Discussion

We investigated serum BDNF concentrations in (a) patients with acute AN-R, (b) individuals long-term recovered from AN-R, both individually age-matched to HCs (cross-sectional arm), and (c) patients with acute AN-R over the course of inpatient treatment (longitudinal arm). In contrast to our hypotheses based on the existing literature [18,32,35], including our own previous work [39,42], we found in the largest known sample to date no significant between-group differences relative to HC or longitudinal changes following partial weight restoration. These null findings were largely corroborated by Bayesian statistics and were further underlined by null results obtained in a post-hoc mega-analysis including data analyzed in our previous study [39]. Importantly, although BDNF levels are known to be susceptible to a range of influencing factors such as age, non-fasting state at blood draw, circadian rhythm, storage time of blood samples [43], sex [14], choice of blood component (i.e., serum or plasma) [77], and physical activity [78], they have not always been rigorously accounted for in previous studies in AN samples [32]. In the current study, we attempted to address these issues by carefully (a) age-matching individuals in the cross-sectional comparisons, (b) collecting blood samples between 7 and 9 a.m. after an overnight fast, (c) only including female participants, (d) controlling for storage time of blood samples and measurement of BDNF concentrations in multiple batches in our statistical analyses, (e) using serum rather than plasma samples to measure peripheral BDNF levels, and (f) exploring the relationship between serum BDNF concentrations and physical activity. Regarding physical activity, it is unlikely that our findings were unduly influenced by acute bouts of exercise before blood samples were drawn because acAN were enrolled in a specialized eating disorder program and patients’ physical activity was restricted and closely supervised by nursing staff. Additionally, effects of long-term physical exercise on basal BDNF levels are thought to be negligible in females [49]. The fact that previous studies have found BDNF alterations to be more pronounced in AN-R than AN-BP [6,33,79] shows the importance of our null findings made in a sample comprised solely of individuals with acute or former AN-R. In light of the null results, it is not surprising that we also found no significant relationships between BDNF concentrations and clinical variables. Together, in contrast to the assumption based on previous findings that decreased BDNF levels in AN may reflect an adaptive mechanism of the undernourished body to increase food intake [34], the results of the current study suggest that BDNF may not be a strong marker of the disorder, either in the acutely underweight state, during weight restoration or after long-term weight recovery.

Although a few other studies on BDNF in AN have also not found significant differences between acAN and HC as in the current study [6,39,61], the majority have reported decreased serum BDNF levels in the underweight state [17,18,32,33,34,36,37,80]. Discrepancies between these previous findings and ours may be due to several factors, including those we attempted to control for (e.g., age, sex, storage time of blood samples) [43]. Our findings of no changes in serum BDNF levels after partial weight rehabilitation also bring needed clarity to heterogeneous results in the few previous longitudinal studies [35,39,40,61] and confirm the previously reported absence of differences in BDNF levels between recAN and HC [39,41,61,80].

In addition to previous findings in AN, it may be worthwhile to consider the current findings in relation to previous studies in other eating disorders. One comprehensive study on serum BDNF concentrations in a sample comprised of individuals with AN, BN and binge-eating disorder (BED) reported decreased BDNF levels in AN and BN, but not in BED [17]. Peripheral BDNF concentrations appear to be generally lower in BN [81] and may recover with therapy [82], but one study found a significant relationship with reward-related learning in a sample of remitted patients [83]. Studies on BDNF in other psychiatric conditions including major depressive disorder [14,84] and anxiety in general, and obsessive-compulsive disorder in particular [16], have similarly reported decreased BDNF levels. Given that depression and anxiety are frequent comorbidities in eating disorders including AN [85,86], decreased serum BDNF levels in AN and BN could at least be partially accredited to comorbidity. However, we assessed depressive symptoms, which were elevated in all AN groups, in our current study and did not observe any significant relationship between BDNF levels and depressive symptoms.

Animal models have shown that BDNF deficient mice exhibit obesity and hyperactivity [87,88], symptoms that were reversible upon central BDNF infusion [89,90]. This notion is supported by single-case studies on humans with chromosomal aberrations affecting the genes of either BDNF or its receptor Tropomyosin receptor kinase B (TrkB) [91,92]. If these results translate to the pathophysiology of AN, one would generally expect to observe reduced BDNF levels in acAN that increase during weight rehabilitation and normalize after remission, as suggested by the current literature [32,35,41]. On the other hand, whether inferring central BDNF levels from the analysis of peripheral blood samples is appropriate is still subject to an ongoing debate [93,94] and could partly explain the null results from this current study. However, it has been reported that BDNF is able to cross the blood-brain barrier [95], and there is evidence from an animal study that central and peripheral BDNF levels change similarly during developing processes such as aging and maturation [96].

Despite the lack of differences in serum BDNF in our sample, this does not necessarily imply an absence of other meaningful, AN-specific relationships. For example, BDNF in AN has been linked to reward processing [8], the regulation of hedonic eating [33], and psychomotor speed [39] in AN samples. Further, since BDNF is involved in synaptic and brain plasticity [10,11,12,97,98] and the acute state of AN is characterized by remarkable structural brain changes [99,100], it is conceivable to assume a role of BDNF in mediating these alterations, especially given that this relationship has already been described in healthy individuals and other neuropsychiatric disorders [101].

When considering our findings, some limitations apply. First, we only analyzed individuals with acute or former AN-R, so our results might not be generalizable for AN-BP or AN in general. Further, both our acutely underweight and weight recovered samples were on average younger than those included in most previous investigations. A recent study showed that brain atrophy in AN is less readily reversible in older, and therefore more chronic patients [102], and we conversely speculate that alterations in peripheral BDNF concentrations may be more easily detectable in patients with a more chronic clinical course. Additionally, HC were only assessed once, whereas acAN were reassessed after partial weight recovery, which limits insight regarding fluctuations in serum BDNF concentrations between time points at a physiological level. Another potential limitation attributable to the long study duration is the fact that serum samples were measured in three different batches. However, we accounted for potential batch effects statistically. Further, recent findings from dedicated methodological studies [44,103,104] demonstrate an influence of clotting time and temperature on serum BDNF levels, suggesting that even though our protocol for obtaining serum was set out to keep systematic errors to a minimum and is compatible with the majority of other studies in psychiatric populations, serum BDNF levels in our sample may not be readily comparable to results from studies with different protocols. Last but not least, although previous studies have successfully assessed physical activity using the SIAB-EX [54] as in the current study [9,61,62], future studies might employ more comprehensive methods (e.g., electronic devices or physical-activity specific questionnaires) [105].

In conclusion, this current study provides evidence for normal serum BDNF levels in both acAN and recAN of the restrictive type. Although we cannot rule out that BDNF might still be altered centrally in AN and may have important implications on other levels [8,106], our findings seem to suggest that BDNF may have comparatively little clinical relevance compared to other (neuro)endocrine markers, including but not limited to ghrelin [107,108], leptin [109,110], oxytocin [111,112], or gonadal hormones [5,113]. Future studies in AN samples might be better advised to focus on these and other candidate hormones regulating food intake as well as their complex interactions. Future research in this direction might prove beneficial towards developing novel therapeutic targets in AN [5,114,115].

## Figures and Tables

**Table 1:** Cross-sectional study sample: demographic and clinical characteristics.

	*N*	acAN	HC_acAN_	t/W	*p*	recAN	HC_recAN_	t/W	*p*
Age (years)	77/77/62/62	16.5 ± 3.3	16.5 ± 3.3	<0.01	0.996	22.2 ± 3.5	22.2 ± 3.5	−0.01	0.99
IQ	69/77/62/61	113.6 ± 12.3	112 ± 9.2	−0.89	0.376	110.2 ± 9.4	110.1 ± 9.5	−0.06	0.955
BMI (kg/m²)	77/77/62/62	14.9 ± 1.4	20.6 ± 2.2	19.19	<0.001	20.8 ± 1.8	21.8 ± 2.2	2.68	0.008
BMI-SDS	77/77/62/62	−3.1 ± 1.3	−0.1 ± 0.7	18.05	<0.001	−0.5 ± 0.6	−0.2 ± 0.7	2.74	0.007
Minimum lifetime BMI (kg/m²)	77/69/62/60	14.4 ± 1.4	19.3 ± 1.7	18.79	<0.001	14.2 ± 1.5	20.1 ± 1.9	18.54	<0.001
BDI-II	76/76/62/61	23.1 ± 10.9	5.7 ± 5.5	−12.45	<0.001	9 ± 8.6	3.9 ± 5.6	−3.88	<0.001
EDI-2 total	72/75/60/61	210.5 ± 46.1	143.3 ± 26.1	−10.82	<0.001	169.5 ± 47.6	134.7 ± 27.4	−4.92	<0.001
EDI-2 Drive for thinness	74/76/61/61	28.1 ± 9.6	13.9 ± 5.5	−11.15	<0.001	20.7 ± 9.1	12.8 ± 4.8	−6.01	<0.001
EDI-2 Body dissatisfaction	75/76/61/61	36.9 ± 11	24.1 ± 8	−8.14	<0.001	31 ± 11.4	23.1 ± 8.2	−4.42	<0.001
EDI-2 Bulimia	74/76/61/61	10.6 ± 4.5	9.5 ± 2.6	−1.77	0.079	10.5 ± 4	9.7 ± 3.1	−1.24	0.219
Physical activity	75/76/61/62	3 (1)	2 (1)	4104	<0.001	2 (1)	2 (1)	2054	0.382

All values are presented as means ± standard deviation with the exception of physical activity which is presented as median (interquartile range) according to the score assigned by administration of the Structured Interview for Anorexia and Bulimia Nervosa (SIAB-EX). Independent samples *t*-tests were used to assess between-group differences in all variables other than physical activity, which was compared using the Wilcoxon/Mann–Whitney U test; t-values, W-values, and *p*-values are reported as test results. At time of blood sample collection, 3/77 acAN and 2/62 recAN received treatment with selective serotonin reuptake inhibitors. Abbreviations: *acAN* acute anorexia nervosa participants; *HC_acAN_* healthy control participants, age-matched to the acAN group; *recAN* long-term recovered anorexia nervosa participants; *HC_recAN_* healthy control participants, age-matched to the recAN group; *IQ* intelligence quotient; *BMI* body mass index; *BMI-SDS* body mass index standard deviation score; *BDI-II* Beck Depression Inventory; *EDI-2* Eating Disorder Inventory-2; *SIAB-EX* Structured Interview for Anorexia and Bulimia Nervosa.

**Table 2:** Longitudinal study sample: demographic and clinical characteristics.

	*N*	acAN-T1	acAN-T2	t/V	*p*
Age (years)	47/47	16.2 ± 2.2	16.4 ± 2.2	−22.88	<0.001
IQ	44	115 ± 12.8	----	----	----
BMI (kg/m²)	47/47	15 ± 1.2	19 ± 1.1	−30.12	<0.001
BMI-SDS	47/47	−2.9 ± 1	−0.7 ± 0.6	−21.29	<0.001
Minimum lifetime BMI (kg/m²)	47	14.8 ± 1.2	----	----	----
BDI-II	46/46	22.1 ± 10.2	12.7 ± 9.9	7.05	<0.001
EDI-2 total	43/43	203 ± 44.3	182.6 ± 46.3	3.52	0.001
EDI-2 Drive for thinness	45/45	26.7 ± 9.8	22.8 ± 10.3	3.18	0.003
EDI-2 Body dissatisfaction	45/45	35.4 ± 11.6	35.1 ± 13	0.11	0.908
EDI-2 Bulimia	44/44	10.5 ± 4.4	8.8 ± 3	2.54	0.015
Physical activity	46/46	2 (1)	1 (1)	510	<0.001

All values are presented as means ± standard deviation with the exception of physical activity which is presented as median (interquartile range) according to the score assigned by administration of the SIAB-EX. Paired-samples *t*-tests were used to assess between-group differences in all variables other than physical activity, which was compared using the Wilcoxon signed rank test; t-values, V-values, and *p*-values are reported as test results. At both study timepoints, 1/47 acAN received treatment with selective serotonin reuptake inhibitors. Abbreviations: *acAN-T1* acute anorexia nervosa participants at study timepoint 1 (admission); *acAN-T2* acute anorexia nervosa participants at study timepoint 2 (after short-term weight rehabilitation); *IQ* intelligence quotient; *BMI* body mass index; *BMI-SDS* body mass index standard deviation score; *BDI-II* Beck Depression Inventory; *EDI-2* Eating Disorder Inventory-2; *SIAB-EX* Structured Interview for Anorexia and Bulimia Nervosa.

## Data Availability

The data presented in this study are not publicly available due to participant data privacy, but anonymized data will be made available on request from the corresponding author.

## Supplementary Materials

The following are available online at https://www.mdpi.com/2072-6643/13/2/432/s1, Figure S1: Serum BDNF concentrations in ng/mL in all study participants plotted over storage time at −80 °C before analyzing. Figure S2: Serum BDNF concentrations in ng/mL in all study participants plotted over squared storage time at −80 °C before analyzing. Figure S3: Results from the Bayesian group comparison of serum BDNF concentrations in the acAN-HC_acAN_ sample. Figure S4: Results from the Bayesian group comparison of serum BDNF concentrations in the recAN-HC_recAN_ sample. Figure S5: Results from the Bayesian group comparison of serum BDNF concentrations in the longitudinal sample. Table S1: Demographic and clinical characteristics of participants recruited from Site A (Berlin) in our previous multi-center study (Cross-sectional). Table S2: Demographic and clinical characteristics of participants recruited from Site A (Berlin) in our previous multi-center study (Longitudinal). Table S3: Correlations between serum BDNF and clinical variables in the original study sample. Table S4: Results from the cross-sectional multiple regression analysis in the combined mega-sample. Table S5: Results from the longitudinal mixed-effects model in the combined mega-sample. Table S6: Results from the cross-sectional multiple regression analysis in the combined mega-sample, excluding all acAN of the binge-purge type (*n* = 17). Table S7: Results from the longitudinal mixed-effects model in the combined mega-sample, excluding all acAN of the binge-purge type (*n* = 2). Table S8: Results from the multiple regression analysis of serum BDNF concentrations between acAN and recAN in the sample of our current study. Table S9: Results from the multiple regression analysis of serum BDNF concentrations between acAN and recAN in the combined mega-sample. Table S10: Results from the longitudinal mixed-effects model in the sample from our current study with age and BMI-SDS as fixed effects. Table S11: Results from the longitudinal mixed-effects model in the combined mega-sample with age and BMI-SDS as fixed effects.
