# Supplementary material for: Is Serum BDNF Altered in Acute, Short- and Long-Term Recovered Restrictive Type Anorexia Nervosa?
Source: Nutrients. 2021 Jan 29;13(2):432. doi: 10.3390/nu13020432 (PMC7910942; doi:10.3390/nu13020432)
Supplement: Supplementary file 1 [file nutrients-13-00432-s001.pdf]

Supplementary Materials

Inclusion criteria and procedures of our previous study on serum BDNF

Inclusion criteria for study participants in our previous work were mostly analog to those in the current study. Nonetheless, inclusion criteria between the two studies did differ in the following aspects: absence of three or more consecutive menstrual cycles was not an inclusion criterion for acAN participants in the current study, because participants were recruited according to the slightly different DSM-V [1] criteria, while individuals in our previous study were recruited according to DSM-IV [2] criteria. However, the vast majority of acAN participants in our current study also presented with amenorrhea. Further, participants from our previous study were assessed within one week after admittance to an eating disorder program, while in our current study participants were assessed within 96 hours after hospital admission. Additionally, participants of the recAN group had to maintain a BMI of 18.5 kg/m^2 or greater (for participants older than 18 years) or above the 10th age percentiles (for participants younger than 18 years) for only 3 months (instead of 6 months) in our previous study. Inclusion criteria for the HC group did not differ between the two studies. In contrast to our previous study, selective psychotropic medication (serotonin reuptake inhibitors) was not an exclusion criterion in the acAN or recAN group in this study and all participants had to refrain from other psychotropic medication or substances for 4 weeks, instead of 6 weeks as in our previous study. A summary of the cross-sectional and longitudinal demographic information of participants from our previous study, who were included in the post-hoc mega-analysis, is provided in Tables S1 and S2.

Collection and processing of blood samples was identical between the two studies, with two exceptions: The time of blood sample collection was between 7:30 and 9:30 a.m. in our previous study and between 7:00 and 9:00 a.m. in our current study. Further, different sandwich enzyme-linked immunosorbent assay (ELISA) kits were used: Our previous study used ELISA kits by Promega Inc. (Madison, WI, USA), adapting to the fluorometric technique [3], whereas our current study used kits by R&D Systems (Minneapolis, MN, USA) according to the standard manufacturer's instructions.

Serum BDNF and storage time

In order to assess the influence of regular and squared storage time on serum BDNF concentrations, these relationships were explored using Pearson correlation. For non-squared storage time, the Pearson's product-moment correlation was negative, significant and of medium strength ($r = -0.20$, 95% CI [−0.31, −0.09], $t(304) = -3.63$, $p < .001$, Figure S1). The Pearson's product-moment correlation between serum BDNF and squared storage time was negative, significant and of small strength ($r = -0.19$, 95% CI [−0.30, −0.08], $t(304) = -3.39$, $p < .001$, Figure S2).

Serum BDNF and clinical variables

We explored the relationship between residualized serum BDNF concentrations and BMI-SDS, EDI-2 total score, BDI-II score, and physical activity using Pearson product-moment correlation (BMI-SDS, EDI-2, BDI-II) and Spearman's rank correlation (physical activity, measured on an ordinal scale). Correlations were calculated for the complete sample as well as for each group separately, pooling HCs from both the acAN-HC$_{acAN}$ and recAN-HC$_{recAN}$ samples. The results are summarized in Table S3.

Results from the Bayesian analysis of serum BDNF concentrations

Results from the traditional NHST indicated no significant group differences in our current sample either cross-sectionally or longitudinally. The strength of NHST lies in the ability to assess the evidence against the null-hypothesis [4]. However, even though the null-hypothesis of no BDNF differences could not be rejected using frequentist analysis, evidence for or against it cannot be assessed using NHST [5].

To overcome this limitation, we carried out Bayesian analyses using JASP [6], using an uniformed Cauchy distribution centered on a zero effect size (δ) and a width of 0.7070 as a prior in all Bayesian analyses. This allowed us to investigate which hypothesis our data supported the best and assess evidence for the null-hypothesis that the cross-sectional and longitudinal study groups do not differ in serum BDNF concentrations [4].

Results from the post-hoc mega-analysis

To verify and extend the results obtained from the sample at the focus of the current study, a supplementary post-hoc mega-analysis was conducted pooling all participants from the current study together with participants from Site A (Berlin) of our previous multicenter study on serum BDNF concentrations in AN [7]. To test for differences in the cross-sectional mega-sample, a multiple regression model with planned contrasts (i.e. acAN vs HC, recAN vs HC) was employed. In the sample examined in this current study, the influence of age on serum BDNF concentrations [8] was addressed by pair-wise matching of acAN and recAN to HC by age. However, this was unfeasible in the combined sample and therefore this effect was controlled for by adding age as an explanatory variable to the multiple regression model. Further, a dummy variable was included in the model, indicating if subjects belonged to the sample of our current or our previous study. Results showed that the cross-sectional groups did not differ significantly on serum BDNF concentrations in the post-hoc mega-analysis (Table S4). Longitudinally, differences in serum BDNF concentrations in acAN before and after partial/short-term weight rehabilitation were assessed using a mixed-effects model allowing to control for the effect of the aforementioned dummy variable. Results indicated no significant differences in serum BDNF concentrations after short-term weight increase in the combined sample (Table S5).

In contrast to the sample at the focus of the current study, we did not limit participants to acAN of the restrictive type in our previous study [7]. Therefore, we repeated our combined-samples analyses after excluding acAN and recAN of the binge-purge type. Nonetheless, results did not change and the differences in serum BDNF concentrations between acAN and HC, recAN and HC and acAN before and after partial/short-term weight rehabilitation remained non-significant (Table S6, Table S7).

Results from confirmatory analyses, excluding participants receiving pharmaceutical treatment

Previous work has suggested that serum BDNF concentrations are influenced by administration of selective serotonin reuptake inhibitors (SSRI) [9]. In our current study, three participants in the acAN group and two participants in the recAN group in the cross-sectional, as well as one participant in the longitudinal comparison received treatment with SSRI within 4 weeks before blood sample collection. To ensure the observed absence of differences in serum BDNF concentrations in all groups was not mediated by receiving treatment with SSRI, we repeated the analyses after excluding all SSRI-medicated participants as well as, for the cross-sectional comparisons, their age-matched HCs.

Nonetheless, the difference of serum BDNF concentrations by group (mean in group HC_{acAN} = 22.23 ng/ml, mean in group acAN = 20.92 ng/ml) remained numerically small and statistically not significant (difference = −1.31 ng/ml, 95% CI [−0.62, 3.26], Welch Two Sample t-test t(145.72) = 1.34, p = 0.182; Cohen's d = 0.22, 95% CI [−0.1, 0.55]).

Similarly, in the recAN-HC_{recAN} sample, the same model (mean in group HC_{recAN} = 21.28 ng/ml, mean in group recAN = 22.11 ng/ml) also revealed a numerically small and statistically not significant group difference (difference = 0.83 ng/ml, 95% CI [−2.77, 1.11], t(117.42) = −0.85, p = 0.397; Cohen's d = −0.16, 95% CI [−0.52, 0.21]).

Results of the longitudinal comparison (mean in group acAN-T2 = 22.24 ng/ml, mean in group acAN-T1 = 20.74 ng/ml) also showed similarly small and statistically not significant differences (mean of the differences = −1.50 ng/ml, 95% CI [−3.11, 0.11], paired t-test t(45) = −1.88, p = 0.066; Cohen's d = −0.28, 95% CI [−0.58, 0.02]).

Controlling for BMI-SDS and age in the longitudinal comparison

To further verify results from the longitudinal comparison of serum BDNF concentrations before and after partial weight-recovery, a linear mixed effects model was administered to both the data of our current sample and the combined sample from our previous investigation of serum BDNF levels in AN [7]. Administration of the linear mixed effects model allowed us to investigate whether controlling for differences of both age and BMI-SDS of participants between the two study timepoints proved to mediate a significant increase of serum BDNF after partial weight-rehabilitation. The differences of both age and BMI-SDS before and after weight-increase were consequently added as fixed effects to the multilevel model. However, previous unsignificant results remained unaffected by this exploratory analysis (Table S8, Table S9).

Quantifying the type II error in the longitudinal comparison

The test result in the longitudinal arm of our main analyses (paired t-test $t(46) = -1.59$, $p = 0.118$) quantifies a probability of observing the test result in our sample, or an even more extreme result, given that the null-hypothesis of no group differences in serum BDNF concentrations across partial weight-rehabilitation is true. Since this result did not meet the widely accepted criterion of a type I error below 0.05 we don't have sufficient evidence to reject the null-hypothesis and therefore the change in serum BDNF across partial weight-recovery cannot be considered statistically significant. However, the aforementioned probability was lower in the longitudinal comparison than in our cross-sectional comparisons and we therefore further quantified the type II error, the probability of incorrectly not rejecting a false null-hypothesis, to validate our results. Since statistical power is the probability of correctly rejecting a false null-hypothesis, the type II error is defined as $\beta = 1 - Power$.

We carried out power-analysis using the "pwr"-package [10] in R [11], assessing the power to detect a medium effect size, as defined by Cohen [12], resulting in a power of 0.919. Therefore, the type II error of incorrectly not-rejecting a false null-hypothesis is 0.081.

Analysis of serum BDNF concentrations between acAN and recAN

Since recAN were significantly older than acAN in our sample (Welch Two Sample t-test $t(127.03) = -9.87$, $p < 0.001$), our approach of pair-wise age-matching participants was not possible for analyzing group differences of serum BDNF concentrations between recAN and acAN. We therefore controlled for possible undue influence of age on serum BDNF concentrations [13] in a multiple linear regression model. To assess differences in serum BDNF levels between recAN and acAN in the post-hoc mega-analysis an additional dummy variable was added to the model indicating if subjects belonged to our current sample or that of our previous study. However, no significant group difference in serum BDNF levels between recAN and acAN could be observed either in the current sample or in the combined sample (Table S10, Table S11).

Table 1. Demographic and clinical characteristics of participants recruited from Site A (Berlin) in our previous multi-center study (Cross-sectional).

	N	acAN	recAN	HC	F	*p*
Age (years)	55/23/52	17.8 ± 3.4 [a],**	20.7 ± 3.8 [b],**	17.7 ± 3	7.56	< 0.001
BMI (kg/m²)	55/23/52	15.1 ± 1.3 [a],**	20.3 ± 1.4 [b],**	21.7 ± 2 [c],**	236.76	< 0.001
BMI-SDS	55/23/52	−3.2 ± 1.3 [a],**	−0.5 ± 0.5 [b],**	0.2 ± 0.7 [c],**	174.78	< 0.001
Minimal lifetime BMI (kg/m²)	5/22/40	14.1 ± 1.5	14 ± 1.6 [b],**	19.4 ± 1.8 [c],**	138.19	< 0.001
EDI-2 Drive for thinness	55/22/52	30.5 ± 9.9 [a],**	15.8 ± 6.4	16.3 ± 7.4 [c],**	45.75	< 0.001

EDI-2 Body dissatisfaction	55/22/51	37.4 ± 11.5 [a],**	26.3 ± 11.2	23.1 ± 8.2 [c],**	20.83	< 0.001
EDI-2 Bulimia	55/22/52	14.2 ± 7 [a],*	9.5 ± 2.8	9.7 ± 3.1 [c],**	11.04	< 0.001

All values are presented as means ± standard deviation. Analysis of variance and post-hoc pairwise comparisons (corrected for multiple comparisons using the Benjamini-Hochberg procedure) were used to assess between-group differences in all variables; F-values and p-values are reported as test results. [a] post-hoc comparison between acAN and recAN [b] post-hoc comparison between recAN and HC [c] post-hoc comparison between acAN and HC * $p < 0.05$ in post-hoc test ** $p < 0.01$ in post-hoc test Abbreviations: *acAN* acute anorexia nervosa participants, *HC* healthy control participants, *recAN* long-term recovered anorexia nervosa participants, *BMI* body mass index, *BMI-SDS* body mass index standard deviation score, *EDI-2* Eating Disorder Inventory-2.

Table 2. Demographic and clinical characteristics of participants recruited from Site A (Berlin) in our previous multi-center study (Longitudinal).

	N	acAN-T1	acAN-T2	t	*p*
Age (years)	14/14	16.6 ± 2.5	16.9 ± 2.8	−4.08	< 0.001
BMI (kg/m²)	14/14	14.6 ± 1.2	17 ± 1.2	−12.43	< 0.001
BMI-SDS	14/14	−3.2 ± 1.2	−1.7 ± 1	−9.87	< 0.001
Minimal lifetime BMI (kg/m²)	14	14.1 ± 1.4	----	----	----
EDI-2 Drive for thinness	14	30.4 ± 10.5	----	----	----
EDI-2 Body dissatisfaction	14	35.9 ± 10.6	----	----	----
EDI-2 Bulimia	14	11.6 ± 6.3	----	----	----

All values are presented as means ± standard deviation. Paired-samples t-tests were used to assess between-group differences in all variables; t-values and p-values are reported as test results. Abbreviations: *acAN-T1* acute anorexia nervosa participants at study timepoint 1 (admission), *acAN-T2* acute anorexia nervosa participants at study timepoint 2 (after short-term weight rehabilitation), *BMI* body mass index, *BMI-SDS* body mass index standard deviation score, *EDI-2* Eating Disorder Inventory-2.

Table S3. Correlations between serum BDNF and clinical variables in the original study sample.

	**BDNF – BMI-SDS**	**BDNF – EDI-2**	**BNDF – BDI-II**	**BDNF – physical activity**
acAN-T1	−0.102 (p = 0.377)	0.097 (p = 0.418)	0.084 (p = 0.471)	−0.138 (p = 0.238)
acAN-T2	0.246 (p = 0.095)	−0.079 (p = 0.599)	0.043 (p = 0.776)	0.039 (p = 0.792)
recAN	0.068 (p = 0.599)	−0.005 (p = 0.97)	0.046 (p = 0.719)	0.022 (p = 0.866)
HC	0.159 (p = 0.082)	0.05 (p = 0.588)	−0.08 (p = 0.386)	0.062 (p = 0.502)
Total sample	0.166 (p = 0.079)	0.002 (p = 0.978)	−0.009 (p = 0.87)	0.043 (p = 0.455)

Pearson (BDNF and BMI-SDS, EDI-2, BDI-2) and Spearman (BDNF and physical activity) correlation coefficients are reported as test statistics along with their corresponding p-values before correction for multiple comparisons. Abbreviations: *acAN-T1* acute anorexia nervosa participants at study timepoint 1 (admission), *acAN-T2* acute anorexia nervosa participants at study timepoint 2 (after short-term weight rehabilitation), *recAN* long-term recovered anorexia nervosa participants, *HC* healthy control participants, *BMI-SDS* body mass index standard deviation score, *EDI-2* Eating Disorder Inventory-2, *BDI-II* Beck Depression Inventory.

Table S4: Results from the cross-sectional multiple regression analysis in the combined mega-sample.

Predictor variables	Dependent variable		
	Serum BDNF concentrations (ng/ml)		
	Estimate (Std. Error)	t	*p*
Contrast: acAN vs HC	−0.434 (0.591)	−0.734	0.463
Contrast: recAN vs HC	0.776 (0.701)	1.108	0.269
Age	−0.009 (0.068)	−0.129	0.897
Included in Zwipp et al. (2014) [7]	−14.662 (0.546)	−26.837	< 0.001
Constant	21.762 (1.330)	16.364	< 0.001
Observations		389	
R^2		0.662	
Adjusted R^2		0.659	
Residual Std. Error		5.013 (df = 384)	
F Statistic		188.168 (df = 4; 384)	

Estimates and their respective standard errors, as well as t-values and p-values for each coefficient are presented. The bottom part of the table shows the number of observations, R-squared, adjusted R-squared, the residual standard error and the F statistic (i.e. parameters to assess the fit of the model). Abbreviations: *acAN* acute anorexia nervosa participants, *HC* healthy control participants, *recAN* long-term recovered anorexia nervosa participants.

Table S5: Results from the longitudinal mixed-effects model in the combined mega-sample.

Predictor variables	Dependent variable		
	Serum BDNF concentration (ng/ml)		
	Estimate (Std. Error)	t	*p*
Group: acAN-T2	1.258 (0.635)	1.982	0.0521
Included in Zwipp et al. (2014) [7]	−14.677 (1.533)	−9.575	< 0.001
Constant	20.928 (0.793)	26.376	< 0.001
Observations	122		

Estimates and their respective standard errors, as well as t-values and p-values for each coefficient are presented, with the total number of observations in the bottom part of the table. Abbreviations: *acAN-T2* acute anorexia nervosa participants at study timepoint 2 (after short-term weight rehabilitation).

Table S6: Results from the cross-sectional multiple regression analysis in the combined mega-sample, excluding all acAN of the binge-purge type (*n* = 17).

Predictor variables	Dependent variable		
	Serum BDNF concentrations (ng/ml)		
	Estimate (Std. Error)	t	*p*
Contrast: acAN vs HC	−0.626 (0.621)	−1.007	0.314
Contrast: recAN vs HC	0.783 (0.709)	1.105	0.270
Age	−0.013 (0.070)	−0.181	0.857
Included in Zwipp et al. (2014) [7]	−14.853 (0.577)	−25.758	< 0.001
Constant	21.892 (1.382)	15.844	< 0.001
Observations	382		
R^2	0.651		
Adjusted R^2	0.647		
Residual Std. Error	5.058 (df = 367)		
F Statistic	171.3 (df = 4; 367)		

Estimates and their respective standard errors, as well as t-values and p-values for each coefficient are presented. The bottom part of the table shows the number of observations, R-squared, adjusted R-squared, the residual standard error and the F statistic (i.e. parameters to assess the fit of the model). No recAN in this sample was of the binge-purge type. Abbreviations: *acAN* acute anorexia nervosa participants, *HC* healthy control participants, *recAN* long-term recovered anorexia nervosa participants.

Table S7: Results from the longitudinal mixed-effects model in the combined mega-sample, excluding all acAN of the binge-purge type (*n* = 2).

Predictor variables	Dependent variable		
	Serum BDNF concentration (ng/ml)		
	Estimate (Std. Error)	t	p
Group: acAN-T2	1.285 (0.654)	1.965	0.0543
Included in Zwipp et al. (2014) [7]	−14.781 (1.656)	−8.925	< 0.001
Constant	20.915 (0.808)	25.879	< 0.001
Observations	118		

Estimates and their respective standard errors, as well as t-values and p-values for each coefficient are presented, with the total number of observations in the bottom part of the table. Abbreviations: *acAN-T2* acute anorexia nervosa participants at study timepoint 2 (after short-term weight rehabilitation).

Table S8: Results from the longitudinal mixed-effects model in the sample from our current study with age and BMI-SDS as fixed effects.

Predictor variables	Dependent variable		
	Serum BDNF concentration (ng/ml)		
	Estimate (Std. Error)	t	*p*
Group: acAN-T2	1.291 (0.81)	1.593	0.118
Δ Age	−11.136 (12.399)	−0.898	0.374
Δ BMI-SDS	−0.535 (1.156)	−0.463	0.646
Constant	24.663 (3.654)	6.75	< 0.001
Observations	94		

Estimates and their respective standard errors, as well as t-values and p-values for each coefficient are presented, with the total number of observations in the bottom part of the table. Abbreviations: *acAN-T2* acute anorexia nervosa participants at study timepoint 2 (after short-term weight rehabilitation), *BMI-SDS* body mass index standard deviation score.

Table S9: Results from the longitudinal mixed-effects model in the combined mega-sample with age and BMI-SDS as fixed effects.

Predictor variables	Dependent variable		
	Serum BDNF concentration (ng/ml)		
	Estimate (Std. Error)	t	*p*
Group: acAN-T2	1.258 (0.6345)	1.982	0.0521
Included in Zwipp et al. (2014) [7]	−14.966 (1.785)	−8.386	< 0.001
Δ Age	−1.391 (4.207)	−0.331	0.742
Δ BMI-SDS	−0.62 (0.951)	−0.652	0.517
Constant	22.656 (2.394)	9.464	< 0.001
Observations	122		

Estimates and their respective standard errors, as well as t-values and p-values for each coefficient are presented, with the total number of observations in the bottom part of the table. Abbreviations: *acAN-T2* acute anorexia nervosa participants at study timepoint 2 (after short-term weight rehabilitation).

Table S10: Results from the multiple regression analysis of serum BDNF concentrations between acAN and recAN in the sample of our current study.

Predictor variables	Dependent variable		
	Serum BDNF concentrations (ng/ml)		
	Estimate (Std. Error)	t	*p*
Group: recAN	0.67 (1.303)	0.515	0.608
Age	0.086 (0.147)	0.582	0.561
Constant	19.592 (2.52)	7.776	< 0.001
Observations	139		
R^2	0.012		
Adjusted R^2	−0.002		
Residual Std. Error	5.822 (df = 136)		
F Statistic	0.854 (df = 2; 136)		

Estimates and their respective standard errors, as well as t-values and p-values for each coefficient are presented. The bottom part of the table shows the number of observations, R-squared, adjusted R-squared, the residual standard error and the F statistic (i.e. parameters to assess the fit of the model). Abbreviations: *acAN* acute anorexia nervosa participants, *recAN* long-term recovered anorexia nervosa participants.

Table S11: Results from the multiple regression analysis of serum BDNF concentrations between acAN and recAN in the combined mega-sample.

Predictor variables	Dependent variable		
	Serum BDNF concentrations (ng/ml)		
	Estimate (Std. Error)	t	*p*
Group: recAN	0.931 (0.864)	1.077	0.283
Age	0.064 (0.1)	0.641	0.522
Included in Zwipp et al. (2014) [7]	−14.187 (0.73)	−19.442	< 0.001
Constant	19.884 (1.78)	11.168	< 0.001
Observations		217	
R^2		0.653	
Adjusted R^2		0.648	
Residual Std. Error		5.094 (df = 213)	
F Statistic		133.3 (df = 3; 213)	

Estimates and their respective standard errors, as well as t-values and p-values for each coefficient are presented. The bottom part of the table shows the number of observations, R-squared, adjusted R-squared, the residual standard error and the F statistic (i.e. parameters to assess the fit of the model). Abbreviations: *acAN* acute anorexia nervosa participants, *recAN* long-term recovered anorexia nervosa participants.

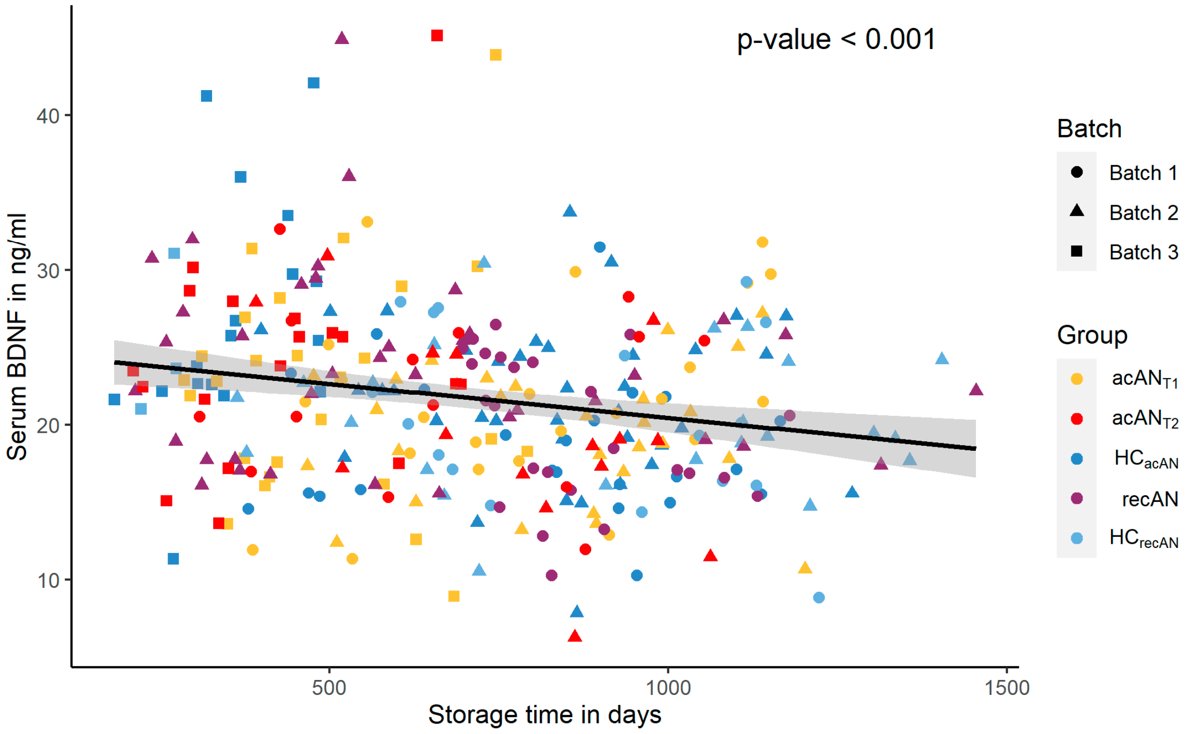

Figure S1: Serum BDNF concentrations in ng/ml in all study participants plotted over storage time at -80°C before analyzing. Batches are represented by different shapes. Study groups are indicated by different colors. The regression line follows the formula of $y \sim x$ and the model is highly significant (p = 0.001). Grey background around the regression line indicates the 95% confidence interval. Abbreviations: *BDNF* brain-derived neurotrophic factor, *acAN$_{T1}$* acute anorexia nervosa participants at study timepoint T1, *acAN$_{T2}$* acute anorexia nervosa participants at study timepoint T2, *HC$_{acAN}$* healthy control participants, age-matched to the acAN group, *recAN* long-term recovered anorexia nervosa participants, *HC$_{recAN}$* healthy control participants, age-matched to the recAN group

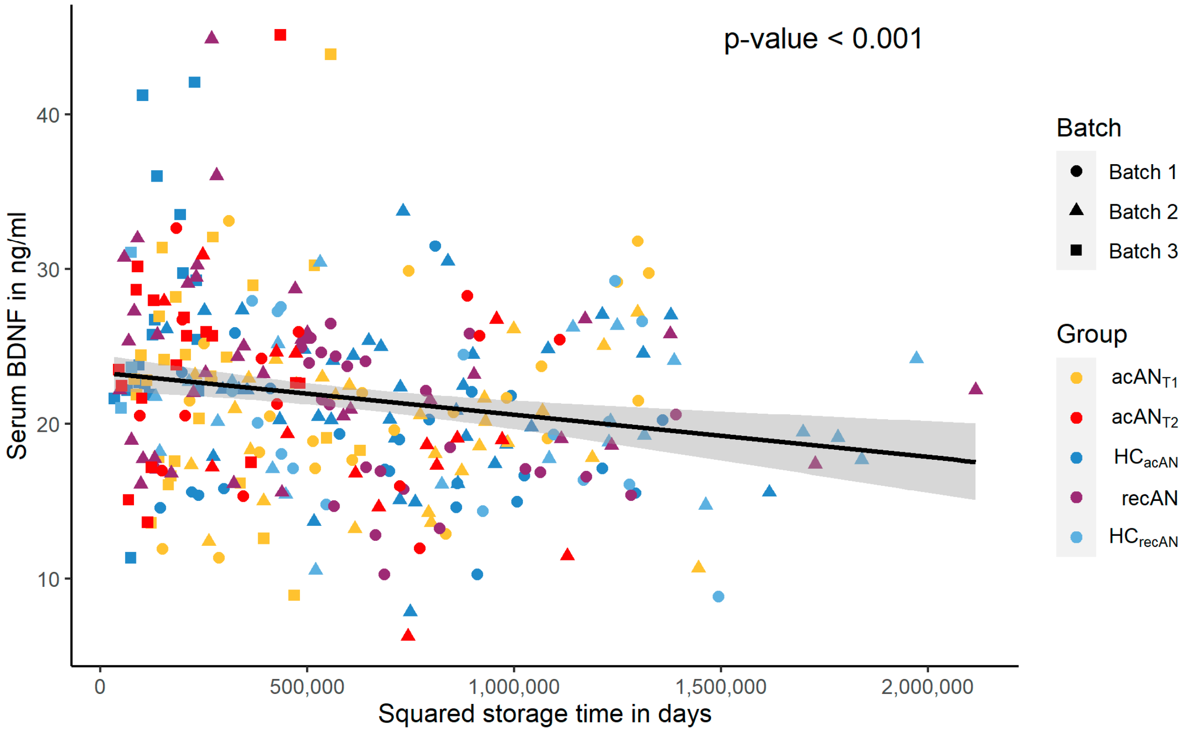

Figure S2: Serum BDNF concentrations in ng/ml in all study participants plotted over squared storage time at -80°C before analyzing. Batches are represented by different shapes. Study groups are indicated by different colors. The regression line follows the formula of $y \sim x$ and the model is highly significant (p = 0.001). Grey background around the regression line indicates the 95% confidence interval. Abbreviations: *BDNF* brain-derived neurotrophic factor, *acAN*$_{T1}$ acute anorexia nervosa participants at study timepoint T1, *acAN*$_{T2}$ acute anorexia nervosa participants at study timepoint T2, *HC*$_{acAN}$ healthy control participants, age-matched to the acAN group, *recAN* long-term recovered anorexia nervosa participants, *HC*$_{recAN}$ healthy control participants, age-matched to the recAN group

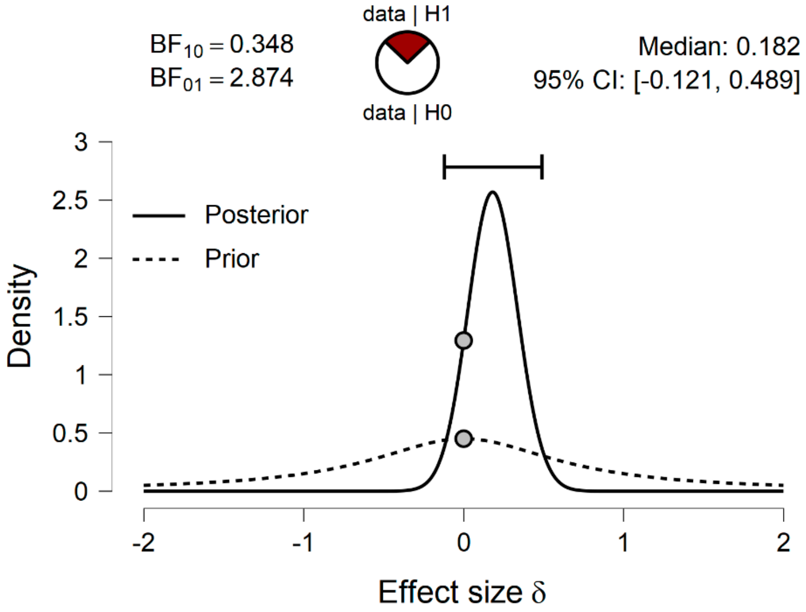

Figure S3: Results from the Bayesian group comparison of serum BDNF concentrations in the acAN-HC_{acAN} sample. The Bayes Factor ($BF_{10} = 0.348$) indicates anecdotal evidence for H_0. The median effect size is 0.182 with a 95% credibility interval (CI) of [−0.121, 0.489].

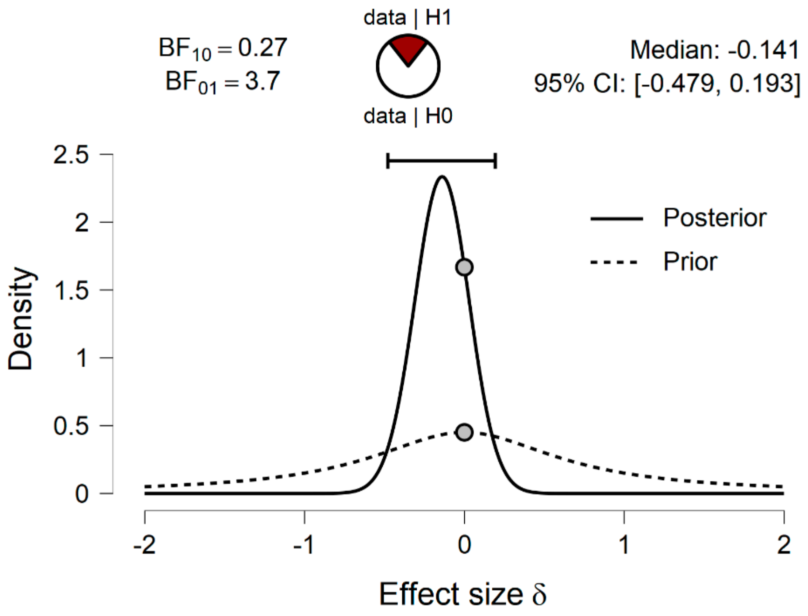

Figure S4: Results from the Bayesian group comparison of serum BDNF concentrations in the recAN-HC$_{recAN}$ sample. The Bayes Factor (BF10 = 0.270) indicates moderate evidence for H0. The median effect size is −0.141 with a 95% credibility interval (CI) of [−0.479, 0.193].

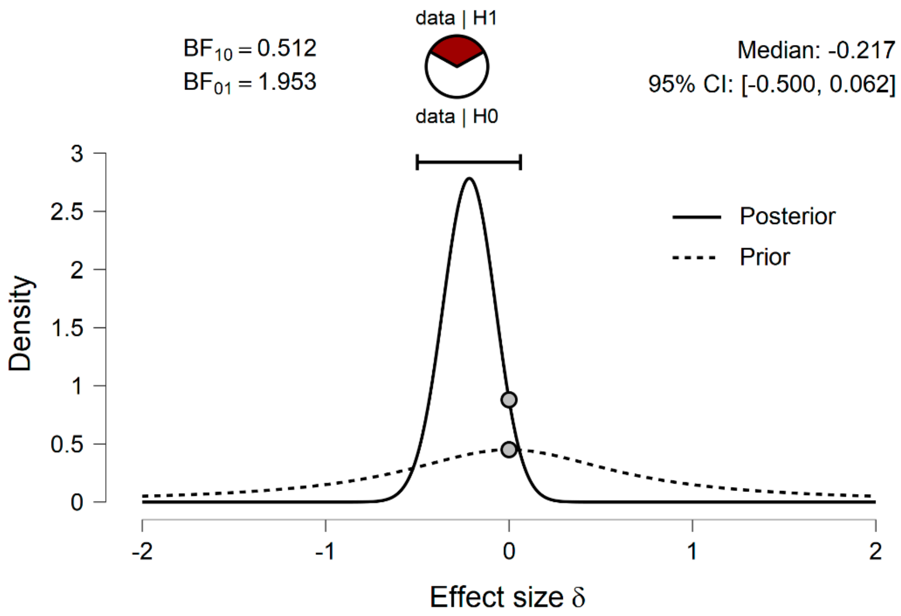

Figure S5: Results from the Bayesian group comparison of serum BDNF concentrations in the longitudinal sample. The Bayes Factor (BF_{10} = 0.512) indicates anecdotal evidence for H_0. The median effect size is −0.217 with a 95% credibility interval (CI) of [−0.500, 0.062].

References used in Supplementary Materials

1. American Psychiatric Association *Diagnostic and Statistical Manual of Mental Disorders*; Fifth Edition.; American Psychiatric Association: Washington, DC, USA, **2013**, ISBN 978-0-89042-555-8.
2. American Psychiatric Association *Diagnostic and Statistical Manual of Mental Disorders, Fourth Edition, Text Revision (DSM-IV-TR)*; 4th ed.; American Psychiatric Association: Washington, DC, USA., **2000**, Vol. 1; ISBN 978-0-89042-334-9.
3. Deuschle, M.; Gilles, M.; Scharnholz, B.; Lederbogen, F.; Lang, U.; Hellweg, R. Changes of Serum Concentrations of Brain-Derived Neurotrophic Factor (BDNF) during Treatment with Venlafaxine and Mirtazapine: Role of Medication and Response to Treatment. *Pharmacopsychiatry* **2012**, *46*, 54–58, doi:10.1055/s-0032-1321908.
4. Keysers, C.; Gazzola, V.; Wagenmakers, E.-J. Using Bayes Factor Hypothesis Testing in Neuroscience to Establish Evidence of Absence. *Nat. Neurosci.* **2020**, *23*, 788–799, doi:10.1038/s41593-020-0660-4.
5. Wagenmakers, E.-J. A Practical Solution to the Pervasive Problems of p Values. *Psychon. Bull. Rev.* **2007**, *14*, 779–804, doi:10.3758/bf03194105.
6. JASP Team. JASP (Version 0.14) [Computer Software]. Available online: https://jasp-stats.org/faq/how-do-i-cite-jasp/ (accessed on 30 October 2020)
7. Zwipp, J.; Hass, J.; Schober, I.; Geisler, D.; Ritschel, F.; Seidel, M.; Weiss, J.; Roessner, V.; Hellweg, R.; Ehrlich, S. Serum Brain-Derived Neurotrophic Factor and Cognitive Functioning in Underweight, Weight-Recovered and Partially Weight-Recovered Females with Anorexia Nervosa. *Prog. Neuropsychopharmacol. Biol. Psychiatry* **2014**, *54*, 163–169, doi:10.1016/j.pnpbp.2014.05.006.
8. Naegelin, Y.; Dingsdale, H.; Säuberli, K.; Schädelin, S.; Kappos, L.; Barde, Y.-A. Measuring and Validating the Levels of Brain-Derived Neurotrophic Factor in Human Serum. *eNeuro*. **2018**, *5*, doi:10.1523/ENEURO.0419-17.2018.
9. Zhou, C.; Zhong, J.; Zou, B.; Fang, L.; Chen, J.; Deng, X.; Zhang, L.; Zhao, X.; Qu, Z.; Lei, Y.; et al. Meta-Analyses of Comparative Efficacy of Antidepressant Medications on Peripheral BDNF Concentration in Patients with Depression. *PLOS ONE* **2017**, *12*, e0172270, doi:10.1371/journal.pone.0172270.
10. Champely, S. Pwr: Basic Functions for Power Analysis; 2020;
11. R Development Core Team *R: A Language and Environment for Statistical Computing*; R Foundation for Statistical Computing: Vienna, Austria, 2019; ISBN 3-900051-07-0.
12. Cohen, J. *Statistical Power Analysis for the Behavioral Sciences*; 2nd ed.; Routledge: Oxfordshire, UK, 2013; ISBN 978-0-203-77158-7
13. Bus, B.A.A.; Molendijk, M.L.; Penninx, B.J.W.H.; Buitelaar, J.K.; Kenis, G.; Prickaerts, J.; Elzinga, B.M.; Voshaar, R.C.O. Determinants of Serum Brain-Derived Neurotrophic Factor. *Psychoneuroendocrinology* **2011**, *36*, 228–239, doi:10.1016/j.psyneuen.2010.07.013.
